# Returning to scientific operations at GM/CA@APS after the APS-Upgrade

**DOI:** 10.1063/4.0001010

**Published:** 2025-10-27

**Authors:** David J Kissick, Michael Becker, Stephen Corcoran, Dale Ferguson, Mark Hilgart, Oleg Makarov, Sergey Stepanov1, Nagarajan Venugopalan, Qingping Xu, Shenglan Xu, Janet L Smith, Robert F Fischetti

**Affiliations:** 1 Argonne National Laboratory, Lemont, IL, USA; 2 University of Michigan, Ann Arbor, MI, USA

## Abstract

The Advanced Photon Source (APS) underwent a comprehensive upgrade to replace the original electron storage ring with a new storage ring (APS-U) that increased the X-ray brightness by up to 500 times compared to the APS. The National Institute of General Medical Sciences and National Cancer Institute Structural Biology Facility at the Advanced Photon Source (GM/CA@APS) operates a national user facility for structural biology. During the year-long shutdown for the APS-U upgrade, the GM/CA beamlines and infrastructure were almost completely rebuilt to exploit the high brightness of the APS-U. New state-of-the-art focusing optics (JTECH Corp. mirrors in Axilon AG benders, and RXOPTICS GmbH and Co. compound refractive lenses (CRLs) in AXILON translocators) now provide an extremely intense (>1013 photons/sec), clean, stable, and rapidly adjustable beam size between 1-50 microns. The maximum energy on 23-ID-D was increased to 35 keV to minimize radiation damage. The new high-stability end station table (Axilon AG) supports both the CRL transfocator and sample environment. The new goniometer allows data collection on crystals down to one micron in size and provides capabilities for rapid scanning of random or periodic fixed target samples. The recently acquired DECTRIS Eiger2 XE 16M CdTe detector enables high-speed, high-efficiency X-ray detection on our high- energy beamline 23-ID-D. The new PyBluIce GUI and beamline control software enables sophisticated routines such as 3D-rastering, helical and fully automated (unattended) data collection, and routine serial crystallography data collection from fixed target and injector- based sample delivery systems. Technical beamline commissioning with the first X-rays began on November 22, 2024, along with macromolecular crystallography measurements of standard samples. On March 11, 2025, we began welcoming users back to participate in scientific commissioning. Here, we will present the new designs and game-changing opportunities for structural biology research enabled by these small, ultra-intense, high-energy beams.

GM/CA@APS has been funded by the National Cancer Institute (ACB-12002) and the National Institute of General Medical Sciences (AGM-12006, P30GM138396). NIH-Office of Research Infrastructure Programs provided funding for the detectors via High-End Shared Instrumentation Grants (Eiger 16M Si- 1S10OD012289-01A1; and Eiger2 Xe 16M CdTe - S10OD034267). This research used resources of the Advanced Photon Source; a U.S. Department of Energy (DOE) Office of Science User Facility operated for the DOE Office of Science by Argonne National Laboratory under Contract No. DE-AC02-06CH11357.

